# Contributive action: socially mediated activities of Russians during the COVID-19 lockdown

**DOI:** 10.1177/1329878X20953536

**Published:** 2020-11

**Authors:** Svetlana S Bodrunova

**Affiliations:** St. Petersburg State University, Russia

**Keywords:** connective action, contributive action, COVID-19, lockdown, Russia, social media

## Abstract

In April to June 2020, Russia passed through a major COVID-19 lockdown which, as elsewhere, has led to a rise of online connectivity and co-practice. We argue that several online mass-participation activities by Russians during the lockdown have grown into examples of *contributive action*. As a type of connective action, contributive action is based upon individual motivation to partake in unorganized personal action. However, its focus moves from connection-and-action (mostly impossible during lockdowns) to online projects where user participation-by-contribution turns an activity into socially and/or politically meaningful action. In Russia, activities based on contributive action, such as virtual protests or online Victory Day celebrations, came in place of connective action at a time when offline collectivity was unavailable or minor. They also served as a way back to normality in relations between the society and the state, as well as a means for social coping with the crisis.

## Introduction

The outbreak of COVID-19 started to affect Russian public life in mid-March 2020. In April and May, all the regions were put under a 40-day lockdown, formally proclaimed as “days off.” Anti-infection policies varied by region, but, by 1 June, only a few regions had eased their anti-coronavirus regime, with Moscow and St. Petersburg only slightly reducing the strictness of their lockdown measures.

As with other locations throughout Europe, the Russian lockdown moved many activities online, from schooling to trade to art. Not only had the formal production of goods and services changed; in many areas, changes were qualitative and new forms of creativity and political action attracted attention much beyond their immediate participants. In this essay, we describe mass socially mediated activities that emerged in Russia during the lockdown and may, we argue, be viewed as examples of *contributive action*; we also reflect upon the role of such activities both as political protest and forms of coping with the crisis.

## “Gimme clear sky”: contributive action in place of connective action

In March to May 2020, several online-only grassroots initiatives of Russians attracted the attention of both domestic and international media. These activities all had in common one distinctive feature. In the circumstances of lockdown, the focus of their participants shifted from *connection-to-action* to *acting by contribution*—adding your own lepta to a bigger project by reproducing someone else’s action that you liked or considered appropriate. We see these projects as based on *contributive action*, an extension to the idea of connective action (Bennett and Segerberg, 2013).

One may argue that, before the lockdown, there had already been many forms of political contributive action. Even liking and sharing, famously negated as “slacktivism” (Gladwell, 2010), might be seen as forms of political contribution, with rather high psychological thresholds to participation in Russia as you can be jailed for online posting. However, what we see as contributive action is not a simple collection of individual contributions. It is a form of collective action seen as such by the society, while still based on individual motivations to provide a contribution. Its value grows from its non-reproducibility offline and an attachment of the act of contributing to distinct online “locations” where a user “puts something”—that is, partaking in contributive action needs “a thing to contribute + an act of contribution.” This, in some cases, presumes a preparatory stage before contribution, while the act of contribution itself appears the same for every user and is technically facilitated. As a result, both the number of people who repeat the same procedure of contribution *and* the contents of the accumulation of contributions start to matter, as they both signal the significance of a given initiative. Although not necessarily initially intended as political, these multiple contributions turn from “activity” to “action” and start to bear a message relevant to the public sphere with a rapid growth in contributive participation and their media profile. Thus, contributive action may be defined as online collective activity based on a massive repetition of an act of contribution which results in a growing body of content with a publicly relevant message.

As with cases of connective action, their formal organizers act simply as facilitators and do not play the most crucial role in how the action grows and spreads. Their original idea matters, but it is participants’ contributions that make this idea turn into (an) action and gain political weight, social relevance, and/or cultural significance.

Earlier forms of online collective action, such as networked flash-mobs or signing online petitions, may also arguably be viewed as contributive action. However, for flash-mobs and petitions, it is the number of people and repetition of the same act that matter, while there is typically no focus on the growing body of content itself. In contrast, in a contributive-action project or initiative, both its essence and social relevance may evolve due to a growth and changes in the accumulated meaning of individual contributions.

Such forms of public activity may become especially relevant when offline circumstances do not allow for the “online-to-offline spillovers” typical of connective action—for example, during the lockdown, but also when autocratic responses to street action may seriously endanger its participants. As the Russian COVID-19 experience shows, contributive action may include projects with clearly political or public memory agendas (with political implications) to those more purely apolitical ones that, as they grow over time, become forms of social coping with a crisis. Below, we describe three examples of such projects and then elaborate on their wider social importance.

### Virtual rallies: a form of contributive protest

During the 2020 lockdowns, several countries have featured street protests against the anti-coronavirus measures. In Russia, the protests initially seemed minor before participants diversified their protest strategies. They protested against the quarantine through disobedience, such as when dozens of drifters partied with prohibited hookahs in St. Petersburg and demanded better management of the crisis, or in Vladikavkaz where offline protests were eventually dispelled by authorities using water cannons.

Russian protesters also developed a form of online-only rallying based on the participation of car drivers. On 20 April in Rostov, drivers started to spontaneously use Yandex.Maps (analogous to Google.Maps, with an option of leaving geotagged comments) to leave protest claims while passing close to the city administration building. Within 24 hours, the rallies spread to at least nine other cities, from Moscow to Siberia. The protesters demanded not only the introduction of a regime of emergency and aid to those affected by COVID-19 but also the resolution of problems unrelated to the pandemic, such as smog (“Gimme clear sky!”) or high commodity tariffs.

The rallies started to receive attention by national media but were interrupted after 2 days as Yandex administrators started to delete the tags. The platform’s publicly announced reason for these deletions was that tags unrelated to traffic conditions were “obstructing the maps” for users. In Moscow, after tags were deleted in the Kremlin area, the “unsanctioned rally” moved to the map of the nearby Tverskaya street—exactly in the manner of an offline rally that runs into a police roadblock. Unorganized *and* un-coordinated, the virtual protests still became a fact of public life.

### The “Immortal battalion online”: a grassroots outburst of war commemoration

The COVID-19 lockdown forced Russian authorities to cancel almost all the 75th World War II Victory Day celebrations on 9 May. This included peaceful rallies of the so-called “Immortal battalion” where people gather to carry the pictures of their dead veteran relatives. The “Immortal battalion” started as a grassroots initiative in 2012 and spread through Russia and abroad. Later, it was largely co-opted into the official celebration discourse and ceremonies and became organized by local administrations, enterprises, and schools. But in 2020, it regained its grassroots nature at 2020.polkrf.ru, VK.com, and YouTube. Within several days, almost 2.4 million users uploaded their pictures of veterans and themselves to these interfaces in a rolling stream, making the “Immortal battalion online,” arguably the biggest lockdown-induced grassroots action in the world. By 1 June, these streams were still live, with new pictures appearing on screen, sometimes weeks after they were uploaded.

In addition to this, hundreds of local communities on VK.com, Russia’s major social network, appeared to be collecting the pictures of local veterans. Accompanied by residents putting veterans’ portraits into windows, while singing the “Victory Day” song at balconies at 7:00 p.m. on 9 May, and many smaller scale activities, the “Immortal battalion online” became the largest substitutive action during the pandemic and a major moment of national grief.

### “Izoizolyacia”: a grateful community of art

An act of creative coping also expressed itself in a Russian Facebook community called “Izoizolyacia.” Its name comes from “isolation” (“***izo****lyacia*” in Russian) and ***izo****brazitel’noe iskusstvo*—“fine art.” Here, reconstructions of visual art objects with improvised materials available at home were published. Initiated on 30 March 2020, the community reached over half a million followers worldwide by 30 April, with hundreds of art objects, from classic fine art to pin-up to film shots, reproduced with the help of people, animals, and interiors, as well as with everyday objects such as potatoes, burnt matches, and toilet paper. Some works reconstructed art pieces with a close attention to detail, some only metaphorically referenced such pieces, some directly referenced the COVID-19 realities while others praised the service of doctors, but many were simply expressions of pure fun.

The first of the isolation art pieces appeared to be a distraction for those burdened by self-isolating with relatives, especially children. For viewers, these expressions apparently raised spirits by demonstrating how unexpectedly creative ordinary families could be—and prompted many to try “isolation art” themselves. These also allowed for expressions of praise for medical workers and play with the realities of COVID-19, making “Izoizolyacia” a (cross-)cultural practice of coping with stress and fear. Here, contributions were not only a form of self-realization but soon became a creative form of social support and expressions of gratitude.

## Contributive action versus the Russian state: a way back to normality?

As noted above, potentially such contributive action may find wide application as an interpretive frame, as these kinds of contribution-based socially mediated activities do not necessarily draw upon clear political goals as their core motivation for individual participants. Our viewpoint is that the three actions—the virtual protest rallies, the Immortal battalion online, and “Izoizolyacia”—were, first and foremost, united by their socio-psychological role, which was, to put it broadly, bringing people back from a period of absurdity to one of common sense and reason. However, as soon as the state became concerned and responded, these initiatives inevitably gained a political dimension.

In a time of low trust to major institutions (Edelman, 2020) and expanding etatism as arguably exists in today’s Russia, the infiltration and intrusion of the state into most areas of public life have been notable. *Sistema* (“the system” of formal and informal ruling entities and practices—see Ledeneva, 2013) that “gardens” critical publics rather than directly represses all dissent (Litvinenko and Toepfl, 2019) has co-opted large areas of free citizenship, from the market economy to public memory. Often, this has meant subjecting the private goals of citizens to those of *sistema*, the latter being perceived as detached from everyday life and even from common sense. Thus, during the pandemic, virtual rallies with their demands of the proper management of the crisis by the authorities have looked like demands for rationality and a disregard of the interests of *sistema*. Admittedly, the appropriateness of such protest in times when an obedience to health safety measures and cooperation with the state has saved lives could be questioned.

If the rallies raised the demand for a normality in decision making, the “Immortal battalion online” returned the country to a normality in grief freed from *ofitsioz*—the officialist slant in bureaucratic discourse, media content, and public events. In turn, “Izoizolyacia” brought their participants and viewers back to irony and optimism, linking individual acts of creativity and an appreciation of culture to the circumstances of the lockdown. The initiative, even if intended ironically, insisted on a continuation of art consumption, a re-addressed appreciation of fine arts, and offered some relief from the stress caused by the COVID-19 lockdown. If “Izoizolyacia” returned its members to a sense of “pre-COVID” normality, the rallies and “Immortal battalion” movements helped regain a feeling of normality in the political domain through attempts to link decision-making and memory practices.

Despite the protest nature of the virtual rallies, all the three initiatives noted above were clearly constructive in their appeal, rather than subversive, oppositional, or denialist. Perhaps this is why they prompted such a large grassroots response. With both “Immortal battalion” and “Izoizolyacia,” user activities spread beyond the Russian borders and even the Russian-speaking online population, demonstrating a high cross-cultural potential for such contributive action.

The significance of prompting a “back to normality” sensation should not be under-estimated, and one sign of it was that the Russian state took their responses seriously. Thus, in Kazan, a woman attracted police attention for the announcement of an online rally on her Facebook page—prompted by new regulations that online rallies now demanded explicit approval from the authorities, in the same manner as offline rallies. Within a week after 9 May, authorities started to re-coopt the initiative by chasing “provocateurs” who sent Nazi leaders’ pictures to the “Immortal battalion online.” Of all the contributive-action-based activities, “Izoizolyacia” was the most fortunate: recognition by the state came after the Pushkin State Museum of Fine Arts announced a competition for “Izoizolyacia” creators. Thus, in each of the three cases, a *cycle of contributive action* included the starting of an initiative by a person or group, its viral spreading, a subsequent rapid growth in participation, and resultant increase in the attention of media, followed by counter-action or co-optation attempts by authorities, and an eventual “plateau” in public attention. The belated reaction by state officials did not alter the major significance and effect that such contributive actions achieved: amid the craziest times around, they provided a taste of normal living.
